# Introductory Address—Rush Medical College, &c.

**Published:** 1861

**Authors:** De Laskie Miller

**Affiliations:** Professor of Obstetrics and Diseases of Women and Children


// CHICAGO

### MEDICAL JOURNAL.

VOL. IV.] October & November, 1861. [Nos. 10 & 11.

### ORIGINAL COMMUNICATIONS.

### INTRODUCTORY ADDRESS, RUSH MEDICAL COLLEGE

By DE LASKIE MILLER, M. D.,
*Professor of Obstetrics and Diseases of Women and Children.*

Gentlemen of the Class:

We have assembled this evening to inaugurate the nineteenth annual course of lectures in Rush Medical College, and the pleasing duty is devolved upon me of extending to you a cordial greeting, and in behalf of my colleagues, the faculty, to bid you welcome.

The pleasure of this duty, is enhanced in no small degree by the relatively large number present on this occasion, of those who have identified themselves with the class, and thereby with the interests of this institution.*

At a time like the present, unparalleled in our national history, when the whole country is distracted by the prevalence of intestine dissensions, and the minds of all classes of our citizens are engrossed by the excitements of the passing hour, or by unpleasant anticipations of the future; it was not expected that any very large proportion of the people, and especially of the youth of our land, would remain indifferent or unmoved by the wildness of the scenes through which we are now passing, so as to turn aside from the broad highway of public commotion, and take their way, unmoved by excite-

* The number of students in attendance at the opening of the current course, exceeded that of any similar occasion since the organization of the College.

ment, in the quiet and peaceful paths of scientific study and investigation.

Youth is emphatically the period of life, when the call of patriotism and the allurements of martial glory are most sure to excite in the breast a prompt response, and incite to deeds of noble daring. The dark cloud of war which now fills the whole national atmosphere, and obscures from view almost every object of ordinary interest, diverts the attention of the student, as it has interrupted the chosen vocation of all classes of society. It requires the change of but a single word, to make the language of the prophetic bard applicable to this country and to the present time:

> "Now all the youth of America are afire
> And silken dalliance in the wardrobe lies,
> Now thrives the armorer; and honor's thought
> Reigns supreme in the breast of every man."

From the effects of causes so potent, in arresting progress in scientific pursuits, and operating adversely to the interests of literary institutions, the friends of Rush Medical College anticipated no immunity. The fact, however, that you, gentlemen, are here this evening as Medical Students, and also that you represent so extensive a portion of our common country, even under the prevailing unsettled condition of public affairs, does not lead to the inference of a want of patriotism on your part; but rather that you are in the direct line of duty; for now, more than ever before, is there a demand for that skill in Medicine and Surgery which it is your aim here to acquire. That your numbers are so much greater than the most sanguine had dared to hope, is a source of great gratification to the faculty, and a reasonable ground for the friends of the institution to felicitate themselves upon its flourishing condition, and upon which they may predicate its future stability and usefulness.

The increasing favor and confidence with which this College is regarded by the profession throughout the country, furnish the stimulus, if other motives were wanting, to incite each member of the faculty to the most lively zeal in advancing the best interests of the classes, which annually convene within its walls, and to unremitting industry to render the course of instruction here, in every department, as complete and perfect as in any institution in the country. The faculty fully appreciate the importance of maintaining in this city, a Medical College of the very highest grade, and possessing, as they do, every facility, and the requisite determination for its accomplishment, the ultimate result may be predicted beyond any contingency.

That the efforts of the present corps of teachers have been received with general satisfaction, is evidenced by the gratifying compliment, conveyed in the rapidly augmenting numbers of each succeeding class in attendance upon the lectures here. Then permit me, in extending to you, gentlemen, our kindly greeting, this evening, to assure you, as I feel at full liberty to do in behalf of my colleagues, that if a predominant interest in your welfare and advancement, manifested by a sedulous discharge of our several duties, may be taken as a guarantee, the present course shall prove even more valuable than any preceding one.

This assurance is based not alone on the individual efforts of the Faculty, but also on the fact that the means of illustration and demonstration at their disposal are so ample, that every subject admitting of it will be presented in a demonstrative manner, or be fully illustrated—the advantages of which cannot be overestimated.

While I speak thus encouragingly of the institution of your choice, gentlemen, I cannot avoid congratulating you upon your selection of a profession. The medical profession is one with which you may well be satisfied. While it commands the respect of all classes of society by its utility, it challenges their confidence no less, by its certainty in securing the highest interests of those who become the recipients of its benefactions. It also exerts a most happy influence upon those devoted to the study of the *science*, or the practice of the *art*, by its tendency to strengthen the mind, refine the affections and elevate the character. It is a mistake to suppose, as many do, that the practice of Medicine, or of *Surgery* even, is calculated to harden the heart, and close the avenues to the kindlier sensibilities of our better nature, for whose words, if not those of the physician, can

> "Dart hope into the soul
> And cause comfort to dawn upon the distressed."

There should be higher and nobler motives to stimulate the medical student to assiduous application in the acquisition of his profession, or the physician to the prompt discharge of his arduous duties, than the mere hope of pecuniary reward, or considerations of social position which the profession promises to confer. Though the first of these may be amply secured, by observing the same rules which ensure it in other departments of human enterprise, viz: by industry, economy and perseverance: and the latter may be as surely attained by the recognition and an honorable observance of the laws of society.

Still these, desirable as they may appear, and even necessary as they are conceded to be, are equalled, if not excelled, by the gratification to be derived from the mental culture which the study of medicine requires, and the pleasurable emotions which follow the conscientious and skillful discharge of the duties of the physician. It is doubtful whether any other vocation demands for its successful practice an amount or variety of knowledge equal to that required for the skillful practice of medicine. In medical knowledge all sciences are represented. There is no branch of learning but may be advantageously applied in the elucidation of some medical principle, or lead to some valuable indication in practice.

The diversity of study has the pleasing effect of keeping the perceptive faculties active, and also of fixing the attention, both essential to progress in every branch of study; while the extent of the field is illimitable, and may engage all the powers of the most gifted and industrious. Thus the pleasure and interest are ever increasing; from the time when the tyro receives his first simple lesson, on through the entire course of his pupilage; nor is the climax reached till the active duties of our noble profession are laid aside at the natural close of life, for, if well employed, the principles which guide the physician are

"The wings wherewith we fly to Heaven."

Should any infer from what I have said, that the pleasures of the physician are unalloyed, and to be enjoyed in listless ease, or exemption from labor and every annoyance, let such be at once undeceived, for I assure you that the life of the physician is one of constant toil, and attended by constant anxiety, from a consciousness of the fearful responsibility assumed. The life of the patient, and his own reputation are depending, and where the event is fatal; by the malignant whispers of malice or envy, he need not be surprised should he occasionally meet with undeserved censure and the most illiberal treatment, especially among the ignorant classes, who, being swayed by prejudice and vulgar errors, judge without reason and condem without mercy. The tongue of slander is as much at liberty as the tongue of truth, nor is it in his power to prevent the former from proclaiming its injurious falsehoods. Since you will hereafter be liable to receive your share of this detraction while in the discharge of the duties which will devolve on you as members of the profession, I may be allowed in passing to give you a general recipe, which will surely antidote all such ills, it is short, and is this: ever act in such a manner as not to deserve it, and the consciousness of having done your duty, will afford you a consolation which nothing can take away.

While some whom I now address, are known to have made considerable progress in the acquisition of medical knowledge and are familiar with the routine of college life; it is reasonable to presume that there are others present on this occasion, who occupy seats in the lecture room as a constituent portion of a medical class, for the first time. With the feelings and emotions of such, I can most fully sympathize. It may be, you feel the diffidence which results from inexperience in the great world, or the loneliness which follows the separation from friends—and the confusion of being surrounded by new and stranger scenes, while you entertain but an imperfect idea of the amount of labor to be performed, or the obstacles to be overcome in the acquisition of the profession, and which profession, you may apprehend, may at last disappoint your hopes, either from your own wants of adaptability for its practice, or for anything which you can affirm to the contrary, by its own inherent elements of uncertainty. All these combined, if allowed an undue influence, will bear so heavily upon the spirit, as to seriously retard your progress toward the goal of ambition. These considerations have suggested to me the propriety of offering some thoughts upon the *certainty* and *utility* of medicine, as a science and an art.

This subject, I am sure, will not be devoid of interest to you, and I deem it a point of the first importance, that all doubt relative to the certainty of medicine should be removed from the minds of those pursuing the study or engaged in the practice of the profession; otherwise success will be at best but problematical. A sincere and true philosophy pervades the words of Cabanis, and they convey a deep sense, which forms the basis of all medical investigation and successful practice, says this philosopher: "In order to study and practice medicine in a proper manner, it is necessary to be impressed with its importance, and to be so impressed we must believe in it." This proposition is so manifest to the reflecting mind as hardly to require elucidation or argument to ensure its general acceptation. The successful cultivation of any department of science, requires on the part of the individual the power to control and fix the attention. The paramount importance of such ability and the disposition to exercise it, must be manifest in the study of medicine which comprises the sum of all sciences. Is it not quite certain that without this faith in medicine, the mind of the student would incline to inactivity and listlessness, and the attention would become so easily diverted, by the thousand distracting influences which always surround him, that but very unsatisfactory progress will be made. This state of mind is equivalent to indifference, and indifference is fatal to true advancement, either in the study or practice of medicine. It will lead to the most serious consequences, in the exercise of the art, by the liability of adopting inefficient or inappropriate means for the removal of disease.

In this, we find the source of much of the odium cast upon the profession, and it is, at least one of the causes of the skepticism, in regard to the certainty of medicine which undeniably prevails to some extent in the public mind. To relieve himself from censure, the unskillful practitioner is ever ready to charge his want of success to the imperfections of medical science. The inconsistency of attributing the failings which result from the incapacity of the individual, to defects of the system needs but be stated to be appreciated.

To assume that the science of medicine is now absolutely perfect, and that every part thereof admits of actual demonstration, would be to argue the impossibility of improvement. While we freely admit the imperfections of the system, we at the same time feel a just pride in affirming, that in no other profession is such rapid progress making towards perfection as in ours. That no class of men are more zealous, nor have they been more successful, in the cultivation of general science, at the same time that they have been engaged in the advancement of their own particular calling, than the members of the medical profession.

It is believed by some that the diversity of opinion which prevails among physicians upon medical topics, theoretical and practical, is an unanswerable argument against the certainty of medicine. But admit this to be valid; and it would be easy to show, that the same charge will hold against almost every other vocation. Do we find a greater unanimity of opinion to prevail among the members of other professions than in our own? It would be interesting in this connection to review some of the peculiarities of the principles and practice of the various vocations, recognized in civilized society, from which we may learn that diversity of opinion is not peculiar to medicine. From the want of time, however, a hasty glance must satisfy, our object not being to cavil at others.

We might naturally suppose that in *Theology* we should find the prevalence of the most perfect harmony of opinion, both upon the great doctrines of Christian faith, and of practice, based as they are upon the word of Inspiration, which is eternal verity, and unchangeable, and so plain, that he who runs may read. And yet, can there be anything more diverse, than the opinions entertained, and drawn from the Holy Scriptures, by those who make the Bible their constant study, and this too, not alone on points of speculation or of inference, but upon what would seem to be the plainest and most positive teaching of the Divine Code.

Or turn we to that other learned profession, the *Law*, which is claimed to be the perfection of human reason, and if it be asserted that the deductions in medicine be in any particular uncertain, what may we not say of those peculiar to the law. Its doctrines are written—its decisions voluminous—and every instinct of man would lead to a just decision, for the whole science is but a question of right and wrong. Here then should we not reasonably expect a concurrence of opinion—an agreement in conclusion, and especially so, as time is taken for mature consideration; and yet the contrary is of such frequent occurrence that the uncertainty of the law has passed into a proverb, which has been sarcastically and pertinently hit by Swift, as follows:

> "Demur, imparlance and essoign
> The parties ne'er could issue join;
> For sixteen years the cause was spun,
> And then stood, where it first begun."

In political economy the same still holds true. Do our statesmen agree in their opinions of manufactures, of banks, of currency, of tariff, or indeed upon any of the great questions of public policy? These instances are sufficient to show, that diversity of opinion is not peculiar to our art, nor does our profession suffer by comparison with others. Navigation so far as it depends upon mathematics, is demonstrative, for by its rules the navigator is at any moment enabled to determine his exact location and the rate of progress. But when a prediction of the duration of the voyage is required, all becomes uncertainty, for a knowledge of the future prevalence of adverse winds is not revealed by his art. And during the prevalence of the tempest,

> "When gathering clouds like meeting armies
> Come on apace,"

And dash the billows in fury high above the prow, threatening destruction to sail and stay, and spar, not only is the suceessful termination of the voyage, but the safety of the bark itself, as much a problem as when the skill of the physician is tested in guiding the frail bark of life, through the struggling elements of disease which threaten its destrnction.

Of the utility conferred by the medical profession upon individuals, upon society and the State, much might be said. These benefits, however, must be estimated by a different standard from that which rules in the ordinary business transactions of life. In these we may approximate if we do not reach with unerring exactness the results of labor. But the utility of the medical profession *cannot* be demonstrated by any pecuniary valuation; it reaches beyond and above all this, and must ever do so, while man remains subject to the same impulses and is actuated by the same motives which now and ever have governed him. The enjoyment of health and the desire for longevity are objects of the most intense interest to all, and only when these can be measured can we fully estimate the value of our profession, for, to secure these, the principles of our art are in constant requisition.

There have been those to question the utility of *science* in medicine, and insist that instinct, unaided by rules, is the best guide, in all cases involving either the health or the life of the individual. To illustrate the absurdity of such an assumption, we need only refer to a few of the ordinary contingencies of ordinary experience. The traveller, under the scorching rays of the meridian sun—way-worn and weary—and parching with thirst, upon reaching the limpid stream that flows from the cool retreat of the mountain recess, would, by the promptings of nature, without delay slake his insatiable thirst, heedless of the danger he would incur. In case of luxation, actuated by the promptings of nature alone, every surgeon knows how strenuously, on account of the pain, the individual would resist the least attempt at motion, though this offers the only means of permanent relief, by the restoration of the joint. Or take another instance: in post partum hæmorrhage, where sudden death is inevitable, except the means of relief be directed by the rules of medical art; or in certain preternatural presentations where two individuals would certainly perish, where it not for well directed efforts to correct the errors of nature, and we surely have sufficient to indicate how futile is the assumption that the principles of medical science are valueless.

You will perceive that the exercise of these principles of medicine, in the prevention or the removal of disease, is something more than a mere trade; for while some of the causes of disease are physical in their nature, and tangible, whose effects may be anticipated, affording us opportunity to neutralize them; others are imponderable, and are everywhere prevalent in nature around us, the perturbations of which in the system are varied almost to infinity. These effects are all the more subtle, as they are manifested in the organized living and ever-changing structures of the body, which are so delicate and inimitable in their arrangement, as to impress the mind most profoundly with awe and admiration. The means to be relied on for the removal of these effects in the system must be regulated by the laws which regulate the sentient-vital principle—which govern the mental manifestations and control the physical forces of the system. The skillful physician must comprehend most perfectly the nature of these laws, and their influence in health and in disease; he must be intimately acquainted with the structure and arrangement of all the organs of the body, so that ike the accomplished musical artist, who, familiar with the office and relative value of every key and chord in the complex structure of his favorite instrument, promptly detects the discord which results from derangements in any part, however slight or seemingly unimportant, and by timely aid restores the normal condition, and thus replaces the discord by the concord of sweetest harmony.

The question, then of paramount importance recurs, what estimate should be placed on the medical profession, and why should it command public confidence? These questions, perhaps, may be best answered by taking a brief retrospect of the profession. That faith is reposed in the efficacy of medicine, is incontrovertible; for, however much individuals may cavil at the profession, while in health, it is their first resort when beset by disease in any of its thousand forms. And what is true of the rofession in this respect at the present time, has been true of it ever since medicine was first cultivated as a science. And that this general conviction in the public mind is well founded, is most fully attested by the records of the past. While history is sometimes our best teacher, it may also be appealed to in support of the right, with the utmost confidence, as its testimony bears the impress of unerring certainty, and by its light, likewise, we are enabled to cast our vision back through the long lapse of centuries, till we observe the origin of our noble science in the early morning of the existence of the race. By such a view we shall be impressed with the fact that medicine did not suddenly reach its present degree of perfection, but gradually, by slow and many times unsteady step, as an illustration taken from early practice will show. The people of high antiquity, exposed the sick in public, so that any who passed, that had been similarly attacked and cured, might give advice for the benefit of the sufferer. And at a later period, this plan was much better calculated to accelerate the progress of the art, for all who were cured of disease, were required to go and make an inscription in the temples, of the symptoms of their disease, and the curative agents which had been beneficial to them, and these registers were kept with the same care as the archives of the nation, and every person had the privilege of going to consult them, and of choosing for his sickness or that of his neighbor, the medicaments of which experience had confirmed the value.

Medicine in this early time occupied a very different position from that accorded to the profession now. Then its influence and efficacy were limited in amount and circumscribed in extent, and as the feeble rays from the glimmering taper seem fitful and uncertain; so in this primitive time was the practice of the healing art. But its office has ever been to cheer and to bless, though feebly and indifferently at first, yet from that time to the present its progress has been steadily onward and upward, till every portion of the civilized world has now become the recipient of its blessings, for, like the glorious luminary at meridian, it dispenses its genial influence upon all.

Medicine relates to nature, its defense and preservation, and no power that man possesses can surpass it in ameliorating the physical condion of the race. The most casual observation is sufficient for the confirmation of this truth, for individual cases cannot be wanting to any, in which they may have seen the efficacy of medicine demonstrated, and which leave on the mind no doubt of its potency. The same thing which you have thus witnessed in your individual observation has been many times repeated in society generally, so that we are enabled to affirm that through the direct influence of the science of medicine, the aggregate duration of human life has been greatly prolonged, even during the last half century. But upon this point, which is one of such inherent importance and so pertinent to the object before us, I will not limit myself to generalities, or mere assertion, but descend to the testimony of figures. In the broad field of vital statistics, we may find incontestible evidence of the verity of our position, and also that by the same influence the duration and the suffering from disease has been greatly abbreviated and alleviated.

The reports of the Parisian Hospitals show, that while, in 1858, one died in seven who were admitted, now only one dies in twelve; thus demonstrating that our science has increased in its ability to save life, in the same order of diseases, and in the same buildings, about seventy-five per cent. in little over half a century.

In other words, in the Paris hospitals, where formerly fourteen men died of each hundred admitted, now only eight die, a saving of six persons in a hundred, and in the eighty thousand who annually pass through these wards, a saving of five hnndred human beings. From the same source we learn that the period of their stay is greatly lessened; the average time of the residence in these institutions was formerly about thirty-nine days, now it is about twenty-four, making a difference that amounts to a very large per centage since 1805.

A corresponding improvement in the treatment of certain special specific diseases, during the same time, is also demonstrated. While in 1805 one patient died in every fifty-six subjected to treatment, now only one case in two hundred and ninety-four proves fatal.

In England, according to Macaulay, the term of human life has been greatly lengthened in the whole Kingdom. In 1685, not a sickly year, one in twenty of the inhabitants died, while at present, in London, only about one in forty dies.

In surgical practice, the saving of life at present exceeds by more than thirty-five per cent. the results at the begining of the present century. The returns of the Registrar General of England show a steady and notable decrease in the rate of mortality. In Renouard's History of Medicine, we read that in France, according to Dapin, the duration of human life has been increasing equal to fifty-two days for each year, from 1776 to 1842, or nine and a half years for the whole period.

The increase per annum was at no period less than nineteen days, although that revolutionary and warlike nation shed seas of blood, not only in her cities, but upon every battle field of Europe.

In obstetric practice, one hundred and fifty years ago, according to Dr. Merriman, *one patient in every forty died.* At the close of his table, in 1828, only one in one hundred and seven died, and at the present time, probably not one in two hundred and fifty.

The improvement in the management of the diseases of infancy is still more conspicuous. M. Quitelet, in his very learned table, states that, in Belgium, of infants born alive, one tenth died within a month, and by the fifth year nearly one half the number of children had died. In Prussia, during the interval from 1820 to 1828, the deaths in the first year were in the ratio of over 26,000 to 100,000. In France, in 1802, it amounted to over 21,000; in Amsterdam, over 22,000, from 1818 to 1829; in Sweden, to over 22,000, from 1821 to 1825. From the Registrar General's report of England, it appears that more than one third of the total deaths in England and Wales occur under one year of age. In Mr. McLean's visit to St. Kilder, he states that eight out of every ten children die between the eighth and twelfth days of their existence. Dr. Combe states that about a century ago, the workhouses of London presented the astounding result of twenty-three deaths in every twenty-four infants under the age of one year; but by improved management, the proportion of deaths was speedily reduced from 2,600 to 450 a year.

One more illustration taken from Dr. Willan's tables, shows the following results:

From 1749 to 1758—1 in 15 children died.
" 1759 to 1768—1 in 20 " "
" 1769 to 1778—1 in 42 " "
" 1779 to 1788—1 in 44 " "
" 1789 to 1798—1 in 77* " "

Much more might be cited to prove, that important and

* Churchill, on Children.

rapid advances have been made in the treatment of infantile diseases, but sufficient has been advanced for our present purpose. By this view of the subject, we are forced to dissent from the sentiment of Spenser, and can only excuse it by granting him a poet's license, when he asserts that,

"The term of life is limited,
Nor may a man prolong or shorten it."

By comparing the hospital reports from the different parts of this country, the same results will be obtained as those derived from corresponding institutions in Europe.

Now, from these various sources, we demonstrate that the duration of human life, through the direct agency of our profession, has been prolonged within the last seventy-five years more than thirty per cent. Should it be urged that the cessation of warlike hostilities, and the general improvement in the condition of the masses, will account for this gain, we have but to compare the reports of hospitals seventy-five years ago, with the reports from the same institutions now, and as these are the peculiar battle fields of the physician, they may with propriety be taken as the indices of what has been done for society at large.

How eloquent are figures in support of the position I have taken! How demonstrative of the utility of our beneficent profession to the best interests of man! It is sometimes interesting thus to take, as it were, a birds-eye view of the profession, as it has made its advances, through the ages past, and note the many triumphs which it has achieved in reaching its present exalted position, and we might, had we time, individualize the persons who have done so much for its perfection and usefulness. Is it probable that a profession which has accomplished so much, and made with rapid strides such a near approach to perfection, is henceforth to stand still, content with what the past has done; or, even as those who put their trust in "pellets" would have us believe, be forced to recede from its present advanced position, loose its hold upon the confidence of an appreciative public, and be lost in forgetfulness? With all this volume and weight of evidence of the utility of medical science, to the State, to society and to individuals, which are accessible to all, and even much more than I have cited, there are those who express a want of confidence in medicine as taught and practiced by the profession at the present day, but affect a faith in the baseless pretensions of one or other of those excrescences of society, who, attached to some of the irregular and exclusive systems, swarm around, like the flies in the fable, merely to "*bleed*" the victim on whom they may chance to alight, while caught in the branches of credulity. But neither these systems of quackery nor their adherents, or their assumptions, are peculiar to our time. There has never been a period in the history of medicine when there was not one or more opposing sects and exclusive systems, which, by clamoring against the profession, hoped to foist themselves into popular favor; but the history of all such shows how miserably they have failed of ultimate success. Not one of them all, though their name be legion, has endured the ordeal forced upon them by the test of time. Nor was it in the nature of things that they should, for being founded on baseless assumption, it was inevitable that they must succumb to the overwhelming power of *experiment*, *induction* and *demonstration*. *These* constitute the Ithureal spear, at the touch of which they

> "Return
> Of force to their own likeness,
> * * * * * * *
> And start up in their own fiendish shape."

*These* form the Herculean Club that ever has, and must continue to strike off the heads of this hydra monster; *these* constitute the rock that is to bury them successively forever from sight.

That alone is enduring in our profession which is founded upon this rock, *experiment*, induction and *demonstration*. All else, however specious its pretensions, must as inevitably disappear, as the dew before the morning sun. From this stand point, we may safely and confidently predict the fate of the those systems and theories which at the present time endeavor to direct attention from scientific medicine. Is not the history of "*Similia*" and "*infininitesimal dilutions*" already well nigh written? For, what is the practice of the sect to-day? Do they hold to a single cardinal principle of their founder? Let the practice of their leaders answer; they administer the remedy that *experience* has found most efficient, and in doses to produce *sensible* effects. Thus we see there is absolutely nothing left of this system but its name. And why should it not be with this, as with every other assumption which is utterly devoid of sense or reason? If an individual were to claim that he had the power of healing a wound made by a cutlass by extending it with a saber, would you think he could induce followers to exercise faith in his pretensions? Perhaps not; and still there are not wanting those whose opinions upon medicine are quite as absurd as this would be. I am sure I know of no way to account for such a perversion of common sense except it be that their minds are so constituted, that the more you dilute the sense, the stronger to them you make the argument.

You may apply still another test to any or all of the exclusive systems which have been set up in opposition to medicine. While their adherents attempt to cast odium upon the profession, by flippantly asserting that *they* are of the progressive school, while the old school remains where its founders left it, you will ask in vain to have them point out any, even the least real improvement in medicine contributed by their adherents, or that has been introduced as the result of their system. What new fact in Anatomy? what in Physiology or Pathology is indebted to them for its birth and usefulness? What new principle in Therapeutics, or what valuable article of the Materia Medica has been added to the sum of human knowledge, by any of the exclusive systems of the present or past time? I am sustained by the record, when I affirm that nothing absolutely of value has been contributed by any of these pseudo reformers.

Gentlemen, I will not pursue this line of thought further. I am sure you already appreciate, at least in some degree, the importance of the subject, and the extent of attainments which a knowledge of *medical science* requires. If you have a just appreciation either of the extent or variety of learning necessary to constitute you skillful physicians, you cannot fail to perceive that this is to be acquired only by laborious and persevering application. For this purpose you are here, and it is hoped, for the single purpose of preparing yourselves for the discharge of most important and responsible duties. It will be our pleasure, as well as duty, to aid you in so doing. If we may but initiate you into the mysteries of our profession, it is all that we can reasonably hope. We need not attempt to teach you all that you are to learn, for the goal indicating the end of improvement in our profession is not yet in view. This fact should, nay, will stimulate you to extend your explorations, even beyond the regions already surveyed and maped out to guide you in starting. Ever remember that opportunities for the brightest achievements are still before you.

You already infer that I would not have you lapse into a state of listless ease and indifference, vainly hoping that when the period has passed in which we are to meet within these halls consecrated to science, and you have taken your departure and are engaged in the active duties of your calling, that then the needed knowledge will come to you as by intuition. I admonish you now, as I should warn you early, that you may *thus* occupy those seats, till you become transformed into fixtures, and you will still be as far from the attainment of your hopes as ever.

It is related of Mercury, that in exchange for the Lyre, he received from Apollo the "*Caduceus*," the insignia of his power and success. This was a staff, one end of which was ornamented with two wings, and around it two serpents were entwined in graceful coils. The wings were indicative of *diligence*, and the serpents of *prudence*—two characteristics long ago deemed essential to success in every undertaking, and the experience of ages, has fully confirmed the truth of this principle so beautifully illustrated in classic mythology of the ancients.

To you, young gentlemen, just setting out in the attainment of your profession, I know of no more important characteristic than *diligence*, and especially so would you attain an honorable position in the noble profession which you have chosen. If you have undertaken the study of medicine under a deep and abiding appreciation of its responsibilities, and the indispensible necessity of unremitting *diligence* in its pursuit, which I have a right to assume from your presence on this occasion, then I would bid you a hearty God speed in the attainment of your most lofty aspirations.

I feel confident you have resolved, without the least mental reservation, that your industry and application during the current session, shall be commensurate with the imperative demands upon you as medical students. That though the road which you are to travel may lead up the steep ascent, and seem beset with apparently insurmountable obstacles, yet that you will boldly advance. Then let me say, that if you approach the task with a *determined will* and with *diligence*, the obstacles are only apparent, and your ascent will be more and still more easy, as you advance, till what at first seemed tedious and dispiriting, will give you the keenest pleasure, as you hear

> " A voice replying far up the hight,

> *Excelsior !*"

Our position demands that we instruct you not only in the principles of our science, but also what may be essential to some present: that we teach you how to learn. To acquire a knowledge of the principles of medicine while in attendance upon the course of instruction to be given here, which, as in the acquisition of knowledge generally, will require the co-ordination of all the faculties of the mind, and the active and vigilant exercise of all; and not only this, but the co-operation of a healthy body; for if the body be not intact, the mind, from a physical necessity, can neither make the desired or the normal progress, any more than a defective instrument can yield the strains of harmony.

The proper training and exercise of the intellectual faculties hold a place of first importance in your course of education. Observation must be cultivated, perception quickened, memory strengthened, and reflection must be ever active. How can all this be effected? Can it be best accomplished by the practice so universal among medical students, of taking notes of the lectures, during their delivery? These are questions which claim your careful consideration, now, at the opening of the course. Were I to answer from my own experience, or from the observations which I have made upon this particular point, to the latter question, I should unhesitatingly say, no. There are comparatively very few of those who compose the classes in the various Medical Colleges in the country, who can commit the teaching of the lecture to paper with such facility as to omit nothing of real importance. This is of itself an art, that only the few possess who make it the business of their lives. Without disparagement, I think I can state, as the rule, that only disconnected sentences are written down in the lecture room, and these not always the most important. And while this has been thus imperfectly performed, much of the lecture that was of importance to be remembered, is lost beyond recovery; so that at the conclu sion of the hour, but little more than a vague and confused impression of the lecture is left on the mind; and should the student refer to the notes to correct errors or fill up the hiatus, they will be found entirely inadequate, or if a resort be made to the *Text Book* for the purpose, the entire subject must be read, and the lecture, so far as the individual is concerned, need not have been given. But such an amount of reading during the progress of the lectures, is manifestly impracticable. The use of the text book should be limited to recalling the fact or principle inculcated in the lecture, which may accidentally have passed from even a well regulated mind. Is it not clear then that the mode usually followed, of taking notes in the lecture room, is not, in every case at least, calculated to improve the intellectual faculties, or leave a lasting and distinct impression of the subject upon the mind.

I would not have you infer from this, that I disapprove of taking notes of the lectures. The practice, however, which I would recommend to you as worthy a thorough trial, is this: After having given your individual attention to the several lectures of the day, and you have reached your rooms, in the evening, commence with the first lecture given in the morning, and write from memory as much of it as you can; then do the same with the second, and of all the lectures given during the day. Although your first efforts may not be perfectly satisfactory to yourselves, yet, by industry and perseverance, your improvement will be such, that you will, yourselves, be surprised at the progress. I have known those who could thus reproduce the lecture almost entire. When you can do this you will have made the knowledge contained in it your own for all time.

Before passing I wish to impress upon your minds what I conceive to be indispensable, viz: the habit of controlling and *fixing the attention.*

There are good reasons for the opinion that this ability to *fix the attention*, is a principal cause of the difference in the intellectual manifestations of different individuals as we meet them in society. We are in the habit of attributing the cause of such difference to original conformation and natural endowment. While this relieves the individual from much of the responsibility of non-improvement, we still insist that intellectual improvement is to a great extent under the immediate control of the individual, by his voluntary efforts in directing the faculties and fixing the attention. This should be so complete that for the time, by abstraction, to become oblivious to all that would divert the mind or distract the powers of reflection. Except this power be cultivated so as to become habitual, you will subsequently learn, to your great regret, that your time has been well nigh misspent; and that while you have supposed you were making some progress in your studies, you will find that you are but a little way removed from the point whence you set out. For the impressions which are made upon the mind, while in a listless or distracted state are never lasting; they cannot be enduring, but must be as evanescent as

> "The snow-drop in the river,
> A moment white, then fades forever."

This is the result of a physical necessity, depending upon the peculiarity of human organization. The registering ganglia of the brain require that every impression made upon them, be continued for a definite period of time, so that the image may become daguerreotyped there; may be limned by the pencil of the spirit; that memory, aided by this picture, may recall the idea in after time, for use in the active duties of life.

If *diligence* is of the first importance to you as medical students, so is *prudence* essential to ensure your success, not only as physicians, but to you while on the threshold of the profession. Every circumstance which may have any influence upon your individuality either as students or physicians, or as men, claims your most serious consideration, and no circumstance of your lives is to be regarded as of trifling moment, though it have but a seemingly slight influence in giving a bias to your thoughts, or irregularity to your habits. For although it may appear to be slight in itself at the present moment, it may by no means be trifling in its consequences. You are to remember that the human mind is ever plastic, and never more so than in the spring time of life, when it is ready to take any form or move in any direction, which the circumstances of accident or fortune may impress upon it.

Like the waters of the lake, whose mirrored surface is so easily thrown into agitation, that even the dropping of a pebble into its glassy bosom, unsettles its quiet surface; though limited at first, yet the agitation, in widening circles, extends until it is lost in the distance.

So is it with impressions made upon the mind. They may be so slight as well nigh to pass unheeded, but they are ever gaining strength and extending their influence. Therefore I charge you guard most zealously against the admission of all influences, whose *tendency* would be to lead the mind from the practice of industry and the path of virtue and honor. Never for a moment lose sight of your own manliness. You would do well to adopt the sentiment of the poet as your own:

> "We live in deeds, not years—in thoughts, not breaths—
> In feelings, not in figures on a dial;
> We should count time by heart-throbs. He most lives
> Who thinks most—feels the noblest—acts the best."

After what I said, I certainly can have little need to admonish you to guard especially against every inclination, to give loose reins to habits of dissipation or any other vice, the indulgence of which would enervate the intellectual powers, and retard the improvement of the mind. I will not then detain you longer; but, in closing, allow me to reaffirm, that excellence is never granted to man, but as the reward of labor. It argues, indeed, no small strength of mind to persevere in habits of industry, without the pleasure of perceiving those advances, which, like the hands of a clock while they make hourly approaches to their point, yet proceed so slowly as to escape observation. Need I then re-enforce the necessity of continued application. You must have no dependence on your own genius. If you have great talents, industry will improve them. If you have but moderate abilities, industry will supply their deficiency. Nothing is denied to well directed labor—nothing is to be obtained without it.

